# Molecular Engineering of Near-Infrared Fluorescent Probes for Cell Membrane Imaging

**DOI:** 10.3390/molecules28041906

**Published:** 2023-02-16

**Authors:** Shuai Xu, Wenjing Pan, Zhi-Ling Song, Lin Yuan

**Affiliations:** 1 School of Chemistry and Chemical Engineering, University of Jinan, Jinan 250022, China; 2 Shandong Key Laboratory of Biochemical Analysis, College of Chemistry and Molecular Engineering, Qingdao University of Science and Technology, Qingdao 266042, China; 3 State Key Laboratory of Chemo/Biosensing and Chemometrics, College of Chemistry and Chemical Engineering Hunan University, Changsha 410082, China

**Keywords:** cell membrane imaging, small-molecular fluorescent probe, near-infrared imaging, biological application

## Abstract

Cell membrane (CM) is a phospholipid bilayer that maintains integrity of a whole cell and relates to many physiological and pathological processes. Developing CM imaging tools is a feasible method for visualizing membrane-related events. In recent decades, small-molecular fluorescent probes in the near-infrared (NIR) region have been pursued extensively for CM staining to investigate its functions and related events. In this review, we summarize development of such probes from the aspect of design principles, CM-targeting mechanisms and biological applications. Moreover, at the end of this review, the challenges and future research directions in designing NIR CM-targeting probes are discussed. This review indicates that more efforts are required to design activatable NIR CM-targeting probes, easily prepared and biocompatible probes with long retention time regarding CM, super-resolution imaging probes for monitoring CM nanoscale organization and multifunctional probes with imaging and phototherapy effects.

## 1. Introduction

Cell membrane (CM) is the first barrier that separates interior of cells from the extracellular environment and plays a crucial role in physiological processes, such as signal transduction and biomolecular transport [1,2,3,4]. A CM is an amphipathic bilayer membrane consisting of a mix of lipids and proteins [5,6,7]. It is closely related to signal transduction from the extracellular environment, causing further responses through changes in shape and morphology [8,9,10,11]. CM damage can cause cell swelling and apoptosis, eventually causing various diseases, such as cirrhosis, diabetes and even cancers [12,13,14,15]. Therefore, visualization of a CM enables study of related events and evaluation of cell life status and facilitates diagnosis and treatment of CM-related diseases [16,17].

**Scheme 1:**
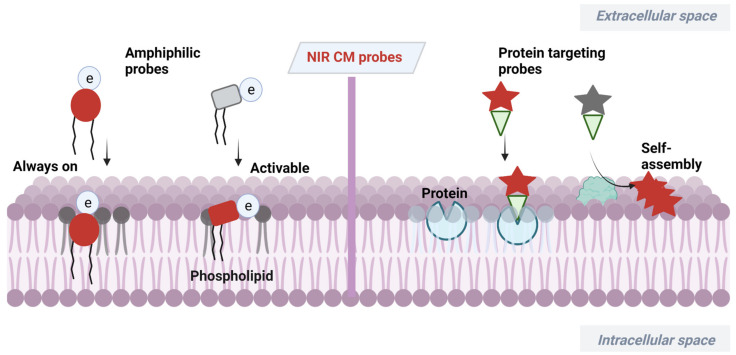
Design strategies of NIR CM-targeting molecular probes.

At present, a certain number of methods have been developed for CM analysis [18,19,20,21]. Among them, fluorescence imaging has become a powerful tool benefiting from its advantages of high sensitivity, simplicity and noninvasiveness [22,23,24,25,26]. Wheat germ agglutinin (WGA) and concanavalin A have served as popular fluorescent CM probes due to their efficiency and ease of use [27,28,29], but these probes are expensive and have large size. Small-molecular fluorescent probes have been widely used for bioimaging due to their easy preparation, modifiable molecular structures and tunable fluorescence signals [30,31,32,33,34]. The small size of this probe enables its precise location in the lipid bilayer, which can serve as a flexible tool for study of CM-related events. Small-molecular fluorescent probes with excitation/emission in the near-infrared (NIR) region (650−900 nm) are much more desirable for bioimaging due to attenuated biological autofluorescence, reduced light damage and increased imaging penetration depth [35,36,37,38]. In recent decades, various NIR CM-targeting molecular probes have been developed for visualization of cell activities, metabolism and cell-to-cell communication on CMs. These probes are commonly designed by incorporating CM-targeting units to NIR fluorophore. In this process, screening of CM-targeting units is very important. Considering the amphipathic characteristics of CMs, designing amphipathic probes that have similarity and intermiscibility to a CM is an effective way to realize CM imaging. NIR fluorophore with large π-conjugation can serve as a lipophilic moiety. In this case, amphipathic CM probes can be obtained by linking hydrophilic moiety, especially positively or negatively charged groups, to NIR fluorophore. Sometimes, long alkyl chains are needed to assist the probe to insert into a CM by hydrophobic interaction with the alkyl chain of a phospholipid [39,40]. In addition to amphipathic characteristics of CMs, many proteins with important biological functions are embedded in CMs. Another approach to realize CM imaging is to design probes that can specifically target these proteins or be self-assembled on the surface of a CM after being activated by these proteins. In this review, we summarize the recent progress of NIR CM molecular probes for visualization of CM-related events (Scheme 1). The unique molecular structure and photophysicality of NIR dyes are fully considered in design and application of CM probes. These CM probes are divided into two classes based on different targeting mechanisms. Their design principles, targeting mechanisms and biological applications are comprehensively overviewed. At the end of this review, the challenges and future research directions in designing NIR CM-targeting probes are fully discussed.

## 2. NIR Molecular Probes for CM Imaging

NIR dyes with large π-conjugate systems usually have strong lipophilicity [41,42]. Owing to the glycerophospholipid bilayer structure of CMs, lipophilic NIR dyes are easily transferred into a lipid bilayer through lipophilic–lipophilic interaction. However, in most cases, lipophilic dye molecules are readily internalized into cells, losing their membrane staining ability. Designing fluorescent probes that mimic the amphiphilic structure of CMs [43] is a feasible way to image bilayer membranes. Many proteins with important biological functions are embedded in CMs of live organisms. Another CM staining approach is to incorporate a membrane proteins targeting group to probes or relying on self-assembly of the probes on a CM after being activated by proteins. Herein, advances in development of NIR fluorescent probes for CM staining have been overviewed, including their design strategies, targeting mechanisms and biological applications.

### 2.1. Amphiphilic NIR Probes for CM Imaging

The amphiphilic NIR CM probe commonly contains hydrophobic moieties to insert into CM through lipophilic–lipophilic interaction and polar headgroups to prevent probe penetrating into cells. The long alkyl chains are sometimes selected to help a probe stay on a CM. Many NIR CM probes retain emission before and after anchoring on the CM, named as “always-on” NIR CM probes. Using these probes, a CM can be stained and the boundary of cells will be observed. However, in this process, repeated washing procedure is required, hindering the real-time and dynamic imaging of CM. To this end, significant effort has been directed at building activatable CM staining probes. One approach to build such probes is to screen for solvatochromic or fluorogenic NIR dyes that are further covalently linked with polar headgroups. These probes have variable emissions (intensity and wavelength), with the environment changed from polar aqueous condition to low polar membrane lipid, thereby achieving CM imaging in an activatable manner. Another approach to construct activatable CM staining probes is relying on disassembly light-up fluorescence strategy. As mentioned above, NIR dyes with large π-conjunction are prone to be aggregated in aqueous solutions, resulting in quenching of their fluorescence [44]. When being inserted into a CM, however, the aggregates are dissolved and dispersed on the CM, leading to significant fluorescence enhancement. In this case, the washing procedure is no longer required because free probes in culture medium are nonfluorescent. Aggregation-induced emission (AIE) dyes are also selected to construct activatable NIR CM probes. These probes are freely rotating in solutions [45], but their intramolecular rotation can be restricted when inserted into a CM, leading to dramatic fluorescence enhancement.

#### 2.1.1. Always-On Amphiphilic Probes

Cyanine dyes can be readily modified to tune desirable properties, including fluorescence wavelength [46,47]. Based on these dyes, several commercially available NIR CM labels, such as DiD and DiR, are obtained by covalently linking with long alkyl chains [48,49]. However, due to their highly hydrophobic nature, DiD and DiR are poorly soluble in aqueous media and always require high concentrations with a large amount of intracellular dotted fluorescence, which renders CM staining very inefficient.

**Figure 1:**
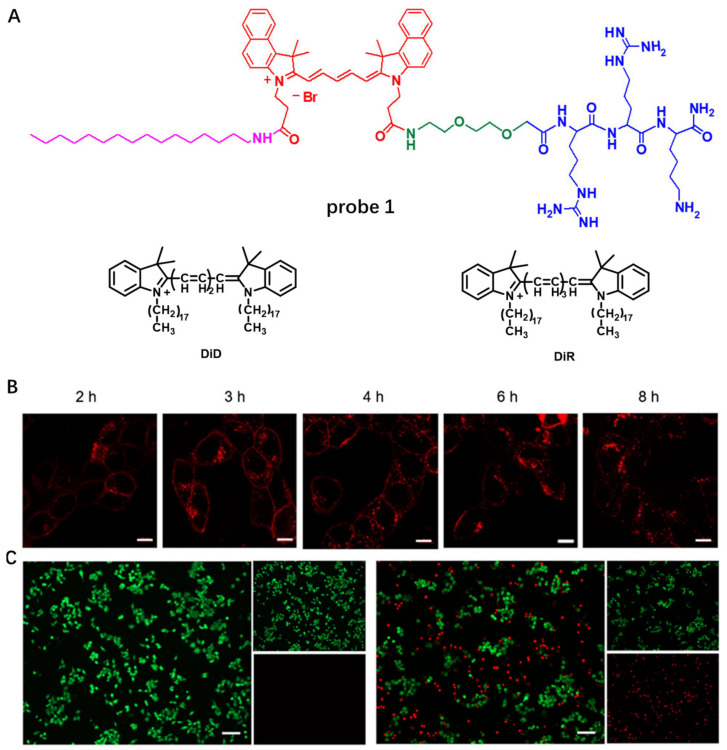
(**A**) Chemical structures of probe **1**, DiD and DiR. (**B**) Live cell imaging using probe **1**. Scale bar: 10 μm (**C**). Photo-thermal ablation of tumor cells with probe **1**. Copyright 2020 Springer Nature [50].

In order to prolong probe retention on CMs and avoid rapid endocytosis, in 2020, Liu et al. developed NIR chimeric peptide probe **1**, which could target tumor CM with long time retention [50]. In probe **1**, Cy5.5 molecule was selected as the NIR dye, Arg–Arg–Lys (RRK) peptide served as a positively charged hydrophilic moiety to target the CM by electrostatic interaction [51], the cetylamine alkyl chain was used to help probe **1** insert into the CM by hydrophobic interaction with alkyl chain of phospholipid [52] and AEEA was applied to increase hydrophilicity and biocompatibility (Figure 1A). Confocal images of 4T1 cancer cells incubated with probe **1** showed that most of probe **1** remained on the CM after several hours, displaying its long retention on CMs (Figure 1B). Moreover, benefiting from its PTT property, probe **1** could exhibit a photothermal effect and destroyed tumor CM in situ and induced cell apoptosis under high-intensity light irradiation (Figure 1C).

**Figure 2:**
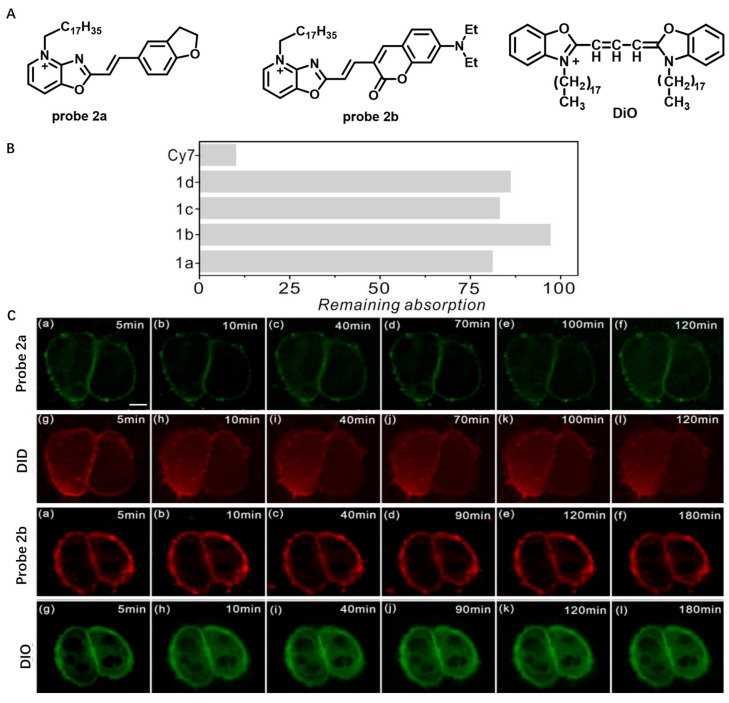
(**A**) Chemical structures of probe **2a** and **2b** and DiO. (**B**) The photostability of probes **2a**, **2b** and Cy7 in acetonitrile. (**C**) Long-term imaging of HeLa CM using probes **2a**, **2b**, DiD and DiO. Scale bar: 10 μm. Copyright 2021 Elsevier [53].

The native properties of cyanine dyes, such as poor photostability and easy aggregation, result in insufficient CM staining for DiD or DiR probes. In 2021, Ge et al. developed several novel NIR CM-targeting probes (**2a** and **2b**) with high optical stability and long retention time based on positively charged oxazopyridine derivatives (Figure 2) [53]. In aqueous solutions, probe **2a** and **2b** hardly aggregated, which was distinct from the commercially available membrane probes (DiD family). Photostability of probe **2a** and **2b** in acetonitrile was also studied using Cy7 as a reference. The results showed that the remaining absorption of probe **2a** and **2b** was more than 80% after 6 h of continuous illumination and less than 10% for Cy7, which indicated that photostability of probe **2a** and **2b** was higher than that of the classical cyanine. The images of HeLa cells using probe **2a** and **2b** displayed that the probes could quickly (<1 min) combine with the CM, and no significant signal change on CM was observed with the culture time increased to 180 min, while CM marker DiD almost completely entered the cells after 10 min. The experimental results showed that probe **2a** and **2b** could rapidly and stably bind to CM.

**Figure 3:**
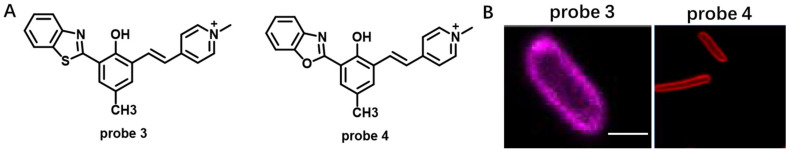
(**A**) Chemical structures of probe **3** and probe **4**. (**B**) Confocal images of prokaryotic cells using probe **3** and probe **4**. Scale bar: 2 μm. Copyright 2018 Elsevier [54] and Copyright 2018 Elsevier [55].

Cyanine dyes always face the problem of small Stokes shift, leading to severe crosstalk between the excitation and emission spectra and low sensitivity of cyanine-dyes-based CM probes. In 2018, Pang et al. developed NIR cyanine probe **3** with a 230 nm Stokes shift by coupling with excited state intramolecular proton transfer (ESIPT) for CM staining of prokaryotic (*E. coli*) cells (Figure 3) [54]. The probe was easily prepared by reacting 2-(2-hydroxyphenyl)benzothiazole (HBT) with pyridinium derivative. Compared to commercial CM dye FM1-43, probe **3** exhibited high fluorescence quantum yield and Stokes shift, enabling probe **3** to be used in a lower concentration. In 2021, using a similar molecular construction strategy, they obtained NIR CM-targeting probe **4** with 260 nm Stokes shift by integrating the ESIPT unit 2-(2-hydroxyphenyl)benzoxazole (HBO) into pyridinium-derived cyanine [55]. Application of this probe in live cells showed that this probe could stain inner CM in prokaryotic cells, *E. coli* (Figure 3).

**Figure 4:**
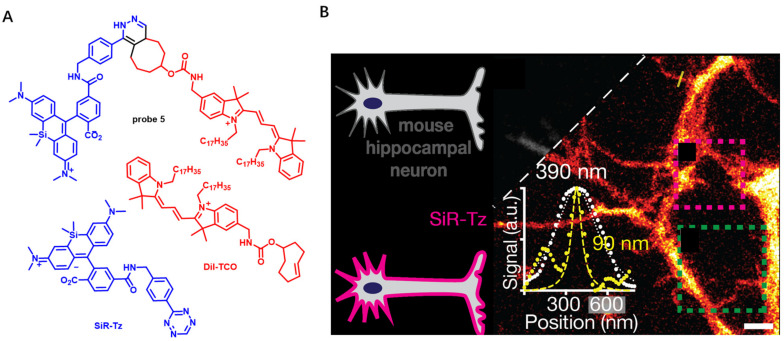
(**A**) Chemical structures of probe **5**, DiI-TCO and SiR-Tz. (**B**) STED imaging of DIV 4 mouse hippocampal neurons with DiI-SiR. Scale bar: 2 μm. Copyright 2017 John Wiley and Sons [56].

Super-resolution microscopy has great advantages in elucidating CM nanoscale organization and quantifying membrane biophysical properties, such as lipid orders. However, poor photostability of current NIR CM probes limits their application in super-resolution CM imaging. In 2017, Schepartz et al. presented a lipid-based strategy to obtain super-resolution images of CM using stimulated emission depletion (STED) (Figure 4) [56]. In this work, high-density-environment-sensitive amphiphilic CM probe DiI-TCO was first applied to stain CM. After a tetrazine ligation reaction, STED dye SiR-Tz could be successfully anchored onto a CM to further produce probe **5**. In this process, robust labelling of CM was clearly observed and individual filopodia could be recognized. In addition, using probe **5**, the STED imaging of primary neurons of central nervous system (CNS) was achieved without the need of transfection (Figure 4B). The above results indicate that probe **5** is able to visualize neuronal dynamics over extended time with high temporal resolution.

**Figure 5:**
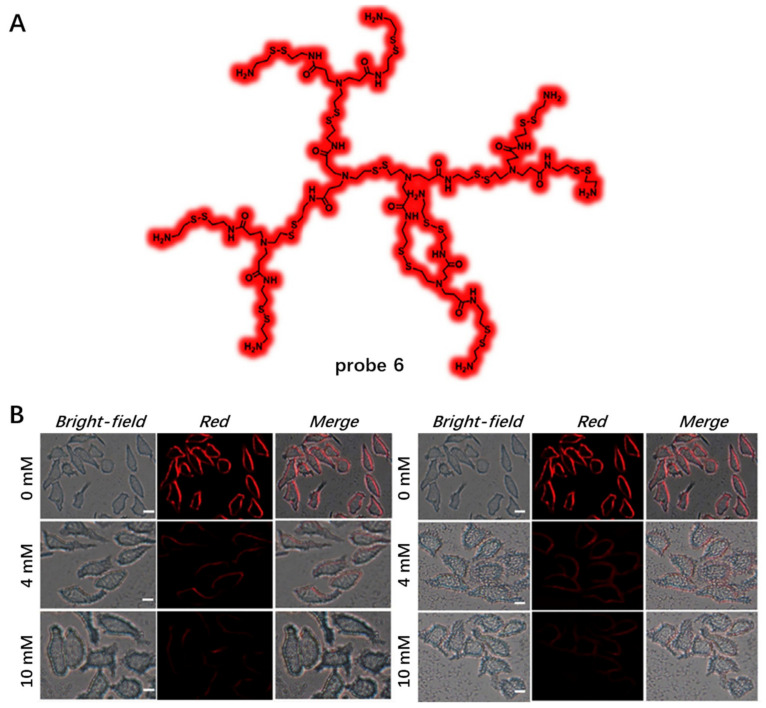
(**A**) Chemical structure of probe **6**. (**B**) CM imaging using probe 6 in the presence of DTT or GSH. Scale bar: 10 μm. Copyright 2022 Elsevier [58].

Compared with NIR dyes bearing large π-conjugated system, fluorescent non-conjugated polymers without any π-aromatic building blocks have great advantages in biological applications, including low cytotoxicity and good biocompatibility [57]. In 2022, Guan et al. developed deep-red/NIR non-conjugated polymer probe **6** with AIE property based on disulfide-linked poly(amidoamine) dendrimers (Figure 5A) [58]. Probe **6** had low cytotoxicity and highly specific targeting ability for HeLa cell CM due to their positive charges on the surface, which could interact electrostatically with the negatively charged surface of the HeLa cell. Upon adding the GSH/DTT to HeLa cells, the red fluorescence signal of probe **6** on CM gradually decreased as probe **6** could be cut off by GSH or DTT (Figure 5B), indicating its good biocompatibility.

#### 2.1.2. Activatable Amphiphilic Probes

##### Solvatochromic- or Fluorogenic-Dyes-Based Probes

Solvatochromic or fluorogenic dyes are sensitive to change in their microenvironment polarity and viscosity [59]. In this case, solvatochromic or fluorogenic dyes are suitable to construct wash-free CM probes pertaining to their weak fluorescence in aqueous solution (high polarity and low viscosity) while strong fluorescence after anchoring on a CM (low polarity and high viscosity). Moreover, solvatochromic or fluorogenic CM probes are useful to reflect the status of CM microenvironment, such as variation in lipid composition, lipid peroxidation and denaturation or conformational change in membrane proteins, by sensing changes in local polarity with different emissions.

**Figure 6:**
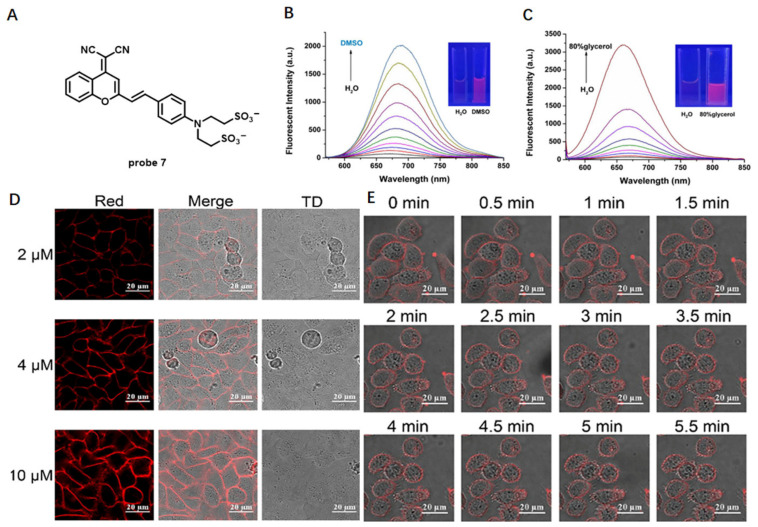
(**A**) Chemical structure of probe **7**. (**B**,**C**) Fluorescent spectra of probe **7** in different conditions. (**D**) HeLa CM imaging using different concentrations of probe **7**. (**E**) The imaging of detachment process of adherent cells using probe **7**. Scale bar: 20 μm. Copyright 2020 American Chemical Society [60].

In 2020, based on a benzene-incorporated dicyanomethylene-4H-chromene derivative (BDCM) with excellent solvatochromic and fluorogenic properties, Zhang et al. developed amphiphilic NIR CM staining probe **7** by linking this fluorophore with two hydrophilic sulfo groups for wash-free imaging of living cells (Figure 6A) [60]. The lipophilic moiety of probe **7** preferred to bring the probe into cells, but the hydrophilic sulfonic group was reluctant to approach the lipids, which finally made this probe stay on CM. In aqueous solution, probe **7** had almost no fluorescence, while its fluorescence intensity at 690 nm increased with addition of DMSO or glycerin, which indicated that probe **7** has excellent solvatochromic and fluorogenic properties (Figure 6B,C). When incubated probe **7** with HeLa cells without washing, fluorescence signals in the red channel were observed on CM, and no fluorescent signal appeared in the nucleus and cytoplasm, which illustrated that probe **7** could specifically stain CM (Figure 6D). Taking this advantage, probe **7** was further successfully used to monitor the detachment process of adherent cells treated with trypsin (Figure 6E). The results indicated that probe **7** is a potential candidate for long-term monitoring of changes in CM morphology and detecting microevents.

**Figure 7:**
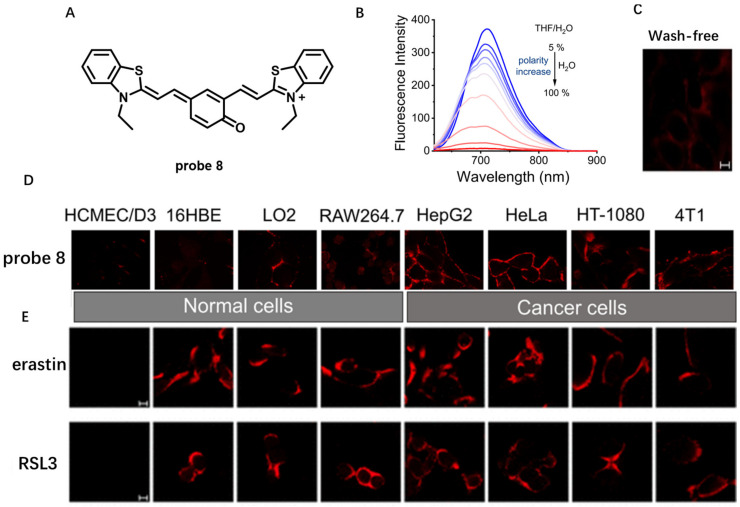
(**A**) Chemical structure of probe **8**. (**B**) Fluorescence spectra of probe **8** in THF/H2O mixtures with different volume proportions. (**C**) Wash-free imaging HeLa cells using 100 nM probe **8**. (**D**) Distinguishing fluorescence imaging of normal cells and cancer cells using probe **8** (λ_ex_ = 561 nm, λ_em_ = 600−700 nm). (**E**) Fluorescence imaging of HT-1080 cells during ferroptosis. Scale bar: 10 μm. Copyright 2022 American Chemical Society [62].

Studies reveal that cancer cells have obviously lower CM polarity compared to normal cells [61]. Therefore, monitoring CM polarity may provide a novel strategy for diagnosis of cancers. In 2022, Li et al. developed polarity-sensitive NIR probe **8** with an amphiphilic molecule structure to monitor microenvironmental polarity in CM (Figure 7A) [62]. Benefiting from its unique D−π−A structure feature, probe **8** showed sensitive fluorescence decrease response at 706 nm with increase in polarity (Figure 7B). Due to its weak fluorescence in aqueous culture medium, specific CM staining in a wash-free manner could be observed even with 100 nM of probe **8**, indicating its efficient CM imaging capability (Figure 7C). In addition, after incubation of probe **8** with four normal and four cancer cell lines, the signals from cancer cells were much stronger than those from normal cells (Figure 7D), indicating that probe **8** could serve as a useful tool for distinguishing normal and cancer cells by monitoring CM polarity difference. Furthermore, using probe **8**, no significant CM polarity variation in the ferroptosis process was observed for the first time (Figure 7E). The above results displayed that probe **8** could be used to monitor polarity variation in CM and may serve as an efficient tool for tumor diagnosis.

**Figure 8:**
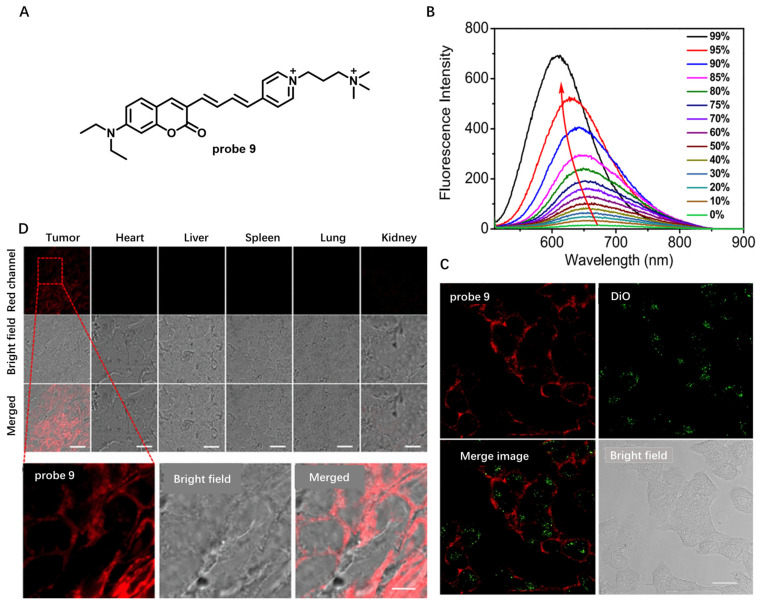
(**A**) Chemical structure of probe **9**. (**B**) Fluorescence spectra of probe **9** in water/1,4- dioxane solvent mixtures. (**C**) Long-term CM imaging using probe **9**. (**D**) Confocal images of tumor tissue and mouse organ tissue sections. Scale bar: 10 μm. Copyright 2022 American Chemical Society [63].

Another work was reported by Feng et al. in 2022 to detect cancer cells and tumors at the CM level (Figure 8) [63]. Probe **9** used in this study was designed as an amphiphilic structure containing a coumarin unit, pyridine salt unit and quaternary ammonium salt unit (Figure 8A). Coumarin was selected as polarity-sensitive dye due to its many advantages, including high fluorescence quantum yield and low cytotoxicity. Pyridine salt was used to prolong the conjugation system and to red-shift the fluorescence of coumarin due to its strong electron-withdrawing properties. A hydrophilic quaternary ammonium salt unit in this probe was able to improve water solubility and increase retention rate in the CM. Probe **9** showed strong fluorescence in low polar solvents and weak fluorescence in highly polar solvents, indicating that probe **9** could respond sensitively to changes in solvent polarity (Figure 8B). Living cell imaging results showed that probe **9** had almost no internalization even after 90 min incubation with HeLa, while DiO showed significant internalization, indicating long-term CM staining of probe **9** (Figure 8C). Using probe **9**, CM imaging in a washing-free manner could be achieved. In addition, probe **9** could specifically light up cancer CM with strong fluorescence in red channel and effectively distinguish tumor tissues from normal tissues (Figure 8D), indicating lower polarity of cancer CMs. This work provided a novel strategy for selective visualization of tumor cells at CM level without using any cancer biomarkers or cancer-cell-specific localization groups.

**Figure 9:**
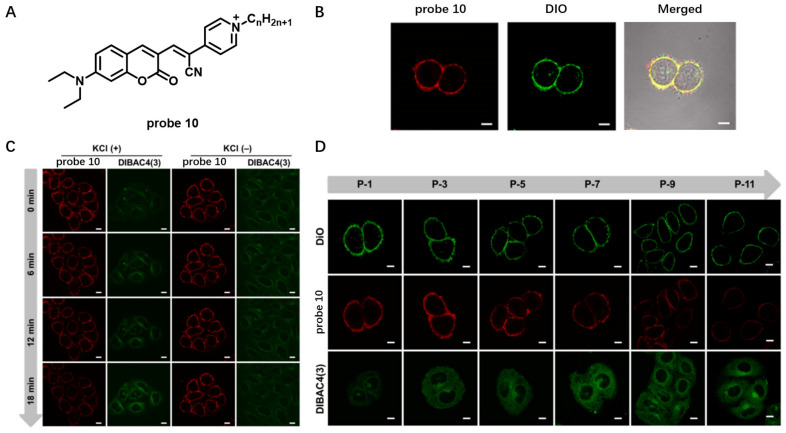
(**A**) Chemical structure of probe **10**. (**B**) CM imaging with probe **10**. (**C**) Fluorescence imaging of the change of ΔΨCM using probe **10**. Scale bar: 10 μm. (**D**) Fluorescence imaging of HeLa cells at different cell passages using probe **10**. Scale bar: 10 μm. Copyright 2020 American Chemical Society [65].

CM potential (ΔΨ) is generated by cells to regulate cellular bioenergetics and propagate signals from excitable tissues to facilitate transmembrane transport of ions [64]. In situ monitoring change in ΔΨ provides a facile way to understand the ion transport mechanism. In 2020, Li et al. developed a coumarin-based cationic probe **10** with NIR emission and large Stokes shifts (Figure 9A) [65]. This probe was built by conjugating (diethylamino)coumarin and cyanopyridinium units, which has strong intramolecular charge transfer (ICT) process. Living cell imaging showed that the CM could be selectively stained by probe **10** in a washing-free manner with a negligible background (Figure 9B). Moreover, probe **10** could sensitively indicate change in ΔΨ adjusted by using KCl solution (Figure 9C), which contributed to its deficient accumulation in the CM once the CM was depolarized with KCl. In addition, this probe was successfully applied to indicate cell passage number of HeLa cells (Figure 9D), and the results showed that, with an increase in cell passage number, the fluorescent intensity of probe **10** gradually became weak, consistent with the study of high-proliferative-state cells accompanied by ΔΨ depolarization.

**Figure 10:**
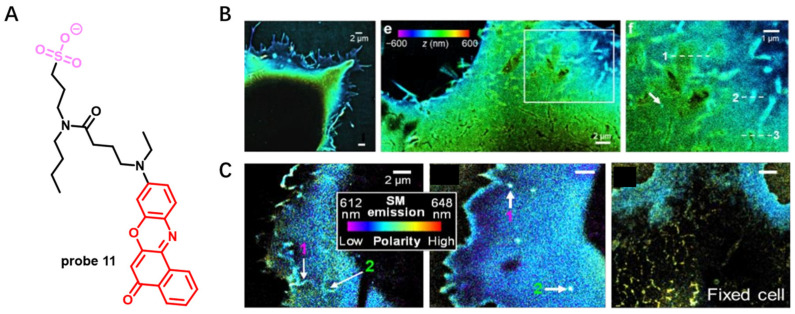
(**A**) Chemical structure of probe **11**. (**B**) 3D PAINT image of COS-7 cells with probe **11**. (**C**) SR-PAINT with probe **11** for CM in live and fixed COS-7 cells. Scale bar: 2 μm. Copyright 2019 John Wiley and Sons [66].

Visualization of the nanoscale organization of CMs with fluorescence probes is now highly pursued by researchers. In 2019, Klymchenko presented a novel strategy for design of super-resolution imaging probe **11** that combined specific CM-targeting, ON/OFF switching and environment sensing functions (Figure 10) [66]. This probe was obtained by connecting solvatochromic dye Nile Red with short alkyl chain bearing an anionic sulfonate group. It served as a promising candidate for points accumulation for imaging in nanoscale topography (PAINT) and used in super-resolution imaging of live cells. Attributed to its high specificity, probe **11** was able to visualize the rich nanoscale structure features of HeLa CM (Figure 10B). In addition, probe **11** was applied to investigate nanoscale distribution of local polarity in CMs. A variation of pseudo-color from blue to cyan of probe **11** was observed in living COS-7 cells (Figure 10C). This work revealed that probe **11**, developed by combination of solvatochromic dye Nile Red and a reversible CM-targeting group, could explore lipid order heterogeneity and nanoscale morphology of CM.

**Figure 11:**
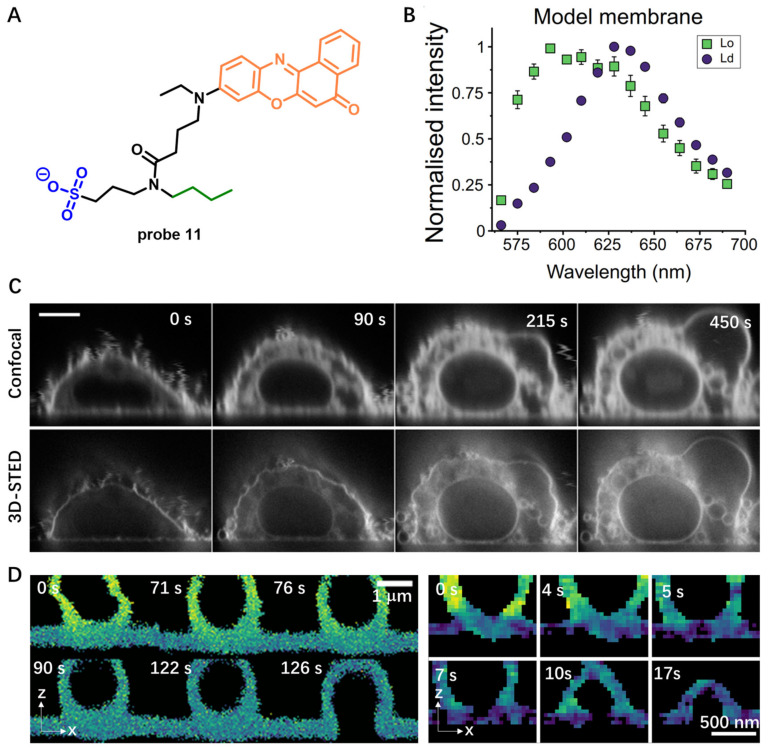
(**A**) Chemical structure of probe **11**. (**B**) The emission spectrum of probe **11** shifts in response to lipid packing. Insert: Lo means liquid-ordered lipid packing, and Ld means liquid-disordered lipid packing. (**C**) Confocal and 3D-STED of CHO cells (up) (Scale bar: 10 μm) or (**D**) model membranes (down) using probe **11**. Copyright 2021 Biophysical Society [67].

In the following work, probe **11** was further used for studying bled formation and lipid exchange during membrane fusion by live-cell real-time 3D STED imaging. Carravilla et al. demonstrated that probe **11** was able to quantify CM biophysical properties for a long time without signal loss due to photobleaching [67]. Emission of probe **11** red shifted by 35 nm after treatment with giant unilamellar vesicles (GUVs) (Figure 11B), showing probe **11** was sensitive to lipid packing variations. Notably, probe **11** was successfully applied for sensing lipid packing variations in live cells using STED microscopy. 3D-STED imaging with probe **11** displayed much higher resolution than confocal images, which enabled visualization of vesiculation and swelling of inner organelles in live cells (Figure 11C) and model membrane systems (Figure 11D). This work highlighted the versatility of probe **11** in confocal and STED imaging applications.

##### Probes Based on Disassembly Light-Up Fluorescence Strategy

Many NIR dyes with large π-conjugated systems are prone to be quenched due to their easy aggregation in aqueous solutions. Disassembly of these aggregates will lead to fluorescence recovery. In this case, disassembly light-up fluorescence strategy is useful to build activatable CM probes. These probes could aggregate in aqueous solution with low fluorescence. After being inserted into the CM, however, the aggregates are dissolved on the CM, leading to significant fluorescence enhancement. Probes that fail to insert into membranes remain off and facilitate wash-free imaging of CM.

**Figure 12:**
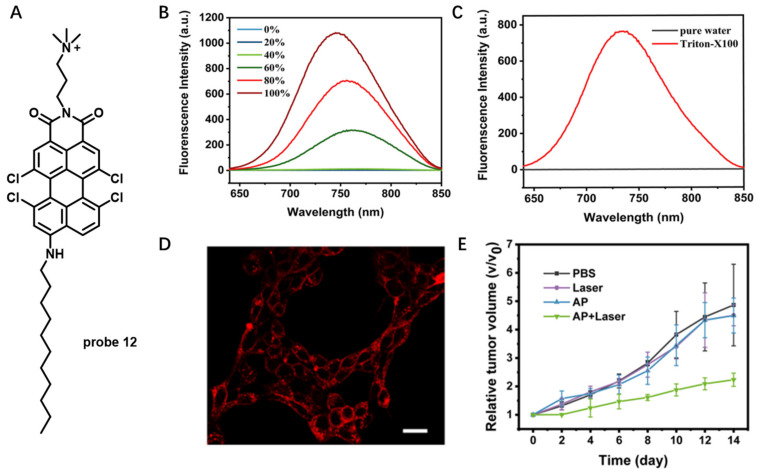
(**A**) Chemical structure of probe **12**. (**B**) Emission spectra of probe **12** in water mixed with increasing percentage of CH_3_OH. (**C**) Emission spectra of probe **12** in the absence or presence of Triton-X100. (**D**) Fluorescence images of CM stained with probe **12**. Scale bar: 100 μm. (**E**) Tumor volume changes of the 4T1 tumor-bearing mice under different treatments. Copyright 2021 American Chemical Society [69].

Perylene molecule with a rigid and planar structure emits strong red fluorescence in organic solvents, and it has poor water solubility and weak fluorescence in aqueous solution due to its easy aggregation [68]. By modifying the structure of perylene molecule, a washing-free CM staining NIR probe will be constructed based on disassembly light-up fluorescence strategy. For example, in 2021, Yu et al. developed an amphiphilic NIR perylene derivative (probe **12**) with a hydrophilic quaternary ammonium functional group and a hydrophobic long alkyl chain (Figure 12A) [69]. In aqueous solution, this probe could self-assemble into nanoparticles without fluorescence emission. Upon addition of CH_3_OH, however, significant fluorescence enhancement at 738 nm was observed, resulting from disaggregation of the nanoparticles in CH_3_OH (Figure 12B). This result clearly indicated that NIR fluorescence emission of the probe **12** was sensitive to the surrounding microenvironment, which was further confirmed by fluorescence enhancement after addition of Triton-X100 into probe **12** solution (Figure 12C). When incubated MCF-7 cells with probe **12** in a wash-free manner, the cell boundaries could be clearly observed without obvious background interference, which made probe **12** a promising probe for wash-free CM imaging (Figure 12D). Probe **12** was also observed to have ^1^O_2_-generation capability. When mice bearing 4T1 tumor were treated with probe **12** + Laser, tumor growth was effectively suppressed compared with the control groups (Figure 12E), which verified that probe **12** was a competent PDT material to suppress tumor growth.

**Figure 13:**
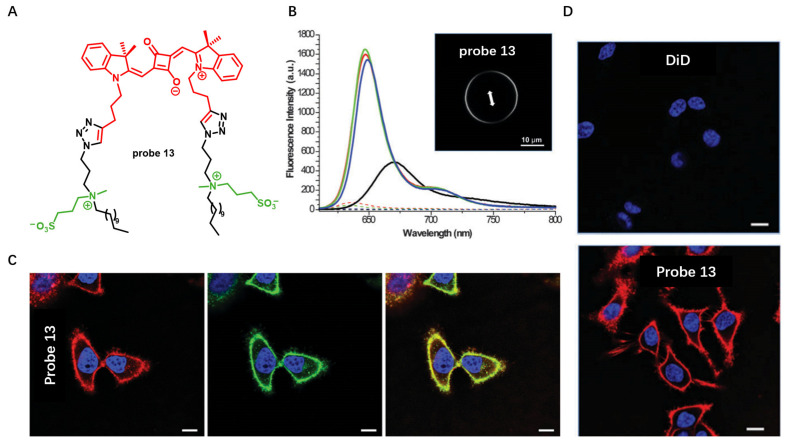
(**A**) Chemical structure of probe **13**. (**B**) Fluorescence spectra of probe **13** in buffer solu-tions and in the presence of lipid membranes. (**C**) Confocal images of CM stained with 1 nM probe **13** without washing. Scale bar: 10 μm. (**D**) CM staining with DiD and probe **13** at 20 nM. Scale bar: 10 μm. Copyright 2015 Royal Society of Chemistry [70].

Squaraine dyes are particularly attractive due to their large π-conjugated systems and long excitation and emission wavelength. In 2015, Klymchenko et al. designed a CM specifically staining probe **13** by incorporating two hydrocarbon chains and two zwitterionic polar head groups to squaraine dye (Figure 13A) [70]. Probe **13** was able to form non-fluorescent aggregates in aqueous buffer while showing strong fluorescence in lipid membranes (Figure 13B). When HeLa cells were incubated with the squaraine probe in a washing-free manner, the CM was specifically stained with exceptional brightness. Notably, compared to the commercial NIR CM probe DiD, probe **13** worked well at 1000-fold lower concentration and stained homogeneously all cells even at nanomolar concentrations (Figure 13C). This excellent property prior to commercial fluorescent plasma membrane probes was largely attributed to replacement of the octadecyl (C-18) chains in DiD with zwitterionic polar head groups.

**Figure 14:**
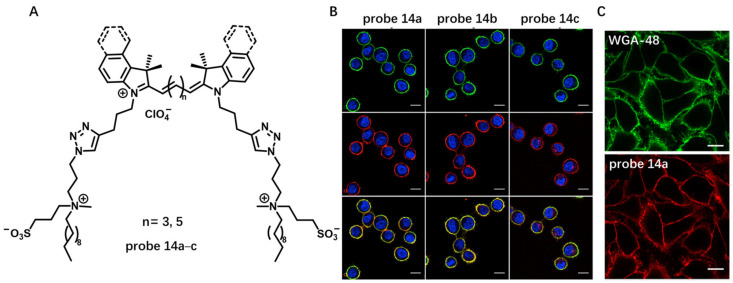
(**A**) Chemical structure of probes **14a**–**c**. (**B**) Confocal images of live cells with probe **14a**–**c**. Scale bar: 15 μm. (**C**) A more homogeneous staining of CM using probe **14a** than using WGA-48. Scale bar: 15 μm. Copyright 2019 Elsevier [71].

In their following work, they applied this design concept to cyanine family to develop several NIR CM probes **14a**–**c** by modifying cyanine dyes with two amphiphilic zwitterionic anchors (Figure 14A) [71]. In aqueous solution, probes **14a**–**c** formed non-fluorescent aggregates, whereas these aggregates dissociated into highly fluorescent molecules in the presence of lipid membranes, which enabled CM imaging without any washing step with high signal-to-noise ratio (Figure 14B). Attributed to their high brightness and efficient CM staining, probes **14a**–**c** were used at very low concentrations (1 nM), which is >1000-fold lower than those commonly used for long-chain cyanines, such as DiD. Moreover, probes **14a**–**c** were able to stain CMs in a more homogeneous manner than the fluorescently labeled WGA (Figure 14C) because the former were small molecules that could diffuse rapidly within the lipid membrane and, unlike WGA, are not dependent on heterogeneous expression of glycan at the cell surface.

**Figure 15:**
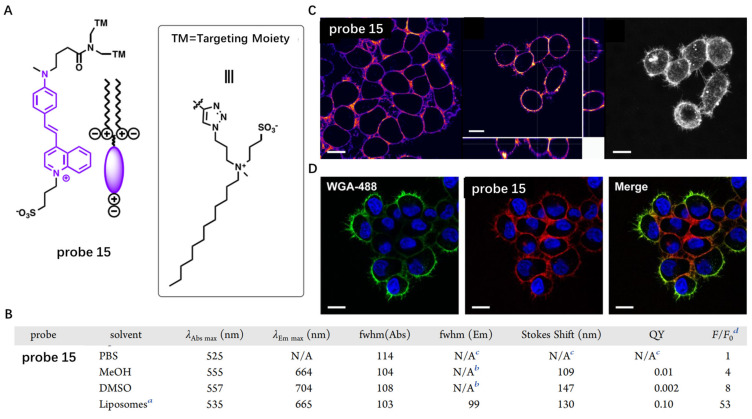
(**A**) Chemical structure of probe **15**. (**B**) The activation of probe **15** in the presence of liposomes. (**C**,**D**) Confocal imaging of live cells stained with probe **15** and commercial probe WGA-488. Scale bar: 15 μm. *^a^* Liposomes (size 100 nm) are composed of DOPC (200 μM final concentration in phosphate buffer 20 mM, pH 7.4). *^b^* The low fluorescence intensity did not allow us to determine the fwhm from the spectrum. *^c^* No proper fluorescence spectrum was obtained. *^d^* Fluorescence enhancement: *F* = Flu-orescence intensity at *λ*_Em_ _max_ in the presence of liposomes, *F*_0_ = fluorescence intensity at the same wavelength in PBS. Copyright 2020 American Chemical Society [72].

These zwitterionic anchors were further used by Klymchenko’s group to modify the structure of commercially available CM probe FM1-43 to obtain novel CM probe (probe **15**) with enhanced photophysical properties (Figure 15A) [72]. The pyridinium moiety of FM1-43 was also replaced by a quinolinium in order to extend its π-conjugation. Probe **15** had weak fluorescence intensity in PBS solutions, while addition of liposomes induced significant fluorescence enhancement at 665 nm (Figure 15B), which resulted from minimized rotation-induced quenching in probe **15**. Living cells imaging showed that probe **15** displayed fast, efficient and selective staining of CM and overlapped well with commercial probe (Figure 15C,D).

**Figure 16:**
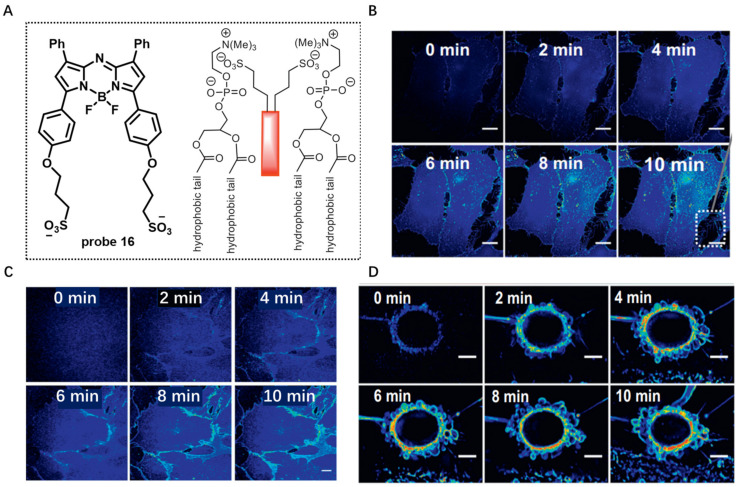
(**A**) Chemical structure of probe **16** and its interaction with membrane bilayer. (**B**,**C**) Live cell imaging in HeLa Kyoto and MC3T3 using probe **16**. Scale bar: 20 μm. (**D**) Real-time image of apoptotic cell with probe **16**. Scale bar: 20 μm. Copyright 2018 Elsevier [74].

BF2-azadipyrromethene fluorophores are widely used for bioimaging because of their excellent photophysical properties, including long wavelength, high quantum yields and photostability [73]. In 2018, based on BF2-azadipyrromethene, O’Shea developed amphiphilic probe **16** by linking two sulfonic acid groups to this dye [74]. The sulfonic acid groups were used to improve water solubility and act as a CM anchor by ionic association with the ammonium component of membrane phospholipids (Figure 16A). The results displayed that the probe was quenched in aqueous solutions due to its aggregation and exhibited strong fluorescence at 717 nm in CH_3_OH due to its disassembly. Living cell imaging in HeLa Kyoto and MC3T3 using probe **16** showed that the CM was quickly lighted up, and many distinctive cellular filopodia protrusions could be observed after 10 min incubation (Figure 16B,C). More interestingly, probe **16** was able to visualize the apoptosis process with increased NIR emission (Figure 16D).

##### Aggregation-Induced-Emission (AIE)-Based Probes

Recently, great efforts have been made to design AIE-based NIR fluorescent probes for CM imaging. When dissolved in solutions, these probes are non-fluorescent due to intramolecular rotation. In contrast, accumulation of these dyes on a CM can lead to dramatic fluorescence enhancement because the intramolecular rotation is effectively restricted, enabling AIE probes ideal candidates for CM imaging.

**Figure 17:**
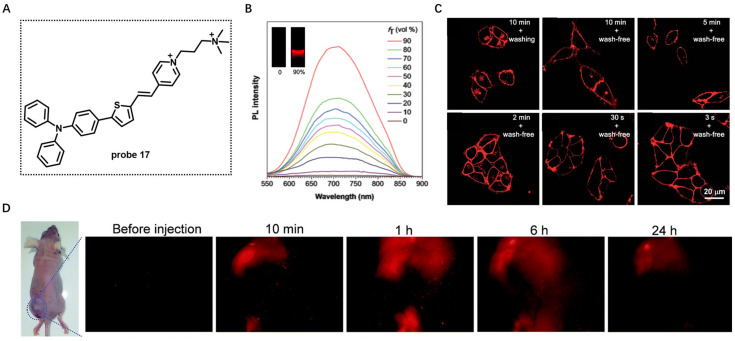
(**A**) Chemical structure of probe **17**. (**B**) PL spectra of probe **17** in water/THF mixtures with different THF fractions. (**C**) Confocal images of living HeLa cells with probe **17** by the use of a washing or wash-free procedure. Scale bar: 20 μm. (**D**) In vivo imaging using probe **17**. Copyright 2018 Royal Society of Chemistry [75].

In 2018, Tang et al. developed water-soluble AIE-based probe **17** with emission in the NIR region for CM imaging [75]. Probe **17** was composed of a triphenylamine moiety (Donor) and a thiophene fragment (Donor and π-bridge), a carbon–carbon double bond (π-bridge) and pyridinium (Acceptor) (Figure 17A). The two positive charges in this molecule conferred high solubility of probe **17** in aqueous solutions. In this case, probe **17** was almost non-emissive in aqueous solutions because the rotational motions of molecular rotors consume exciton energy and increase nonradiative decay rates. When THF was added to this solution, the fluorescence intensity at 708 nm increased and enhanced 97.3-fold at a 90% fraction of THF compared with that of aqueous solutions (Figure 17B). The cell imaging study in HeLa cells showed that the CM was clearly observed with excellent image contrast to the cell background regardless of washing or non-washing process after cell staining with 500 nM of probe **17** for different times (Figure 17C). Surprisingly, the CM was strongly lit up by simply shaking the cell culture with probe **17** for a few seconds at room temperature, showing its ultrafast staining characteristic. In vivo imaging experiment showed that, after intratumoral injection with probe **17** for 24 h, the fluorescence intensity in tumor was still very high (Figure 17D), suggesting outstanding tumor retention properties of probe **17**, possibly benefiting from persistence of membrane insertion.

**Figure 18:**
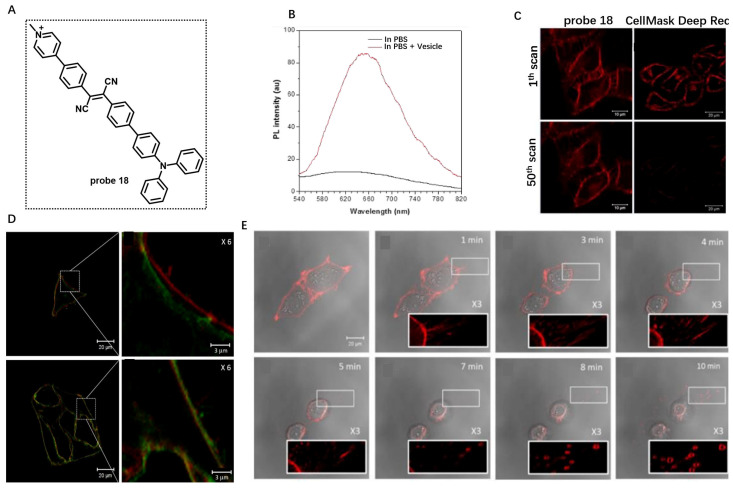
(**A**) Chemical structure of probe **18**. (**B**) PL spectra of probe **18** in the absence or presence of lipid vesicle. (**C**) The photostability of probe **18** in HeLa cells by continuous scanning. (**D**,**E**) Monitoring morphology changes in CM using probe **18**. Copyright 2018 Royal Society of Chemistry [76].

Real-time monitoring of dynamic morphological changes in CM is helpful for biomedical research. In 2018, Tang et al. developed a lipophilic cyanostilbene derivative (probe **18**) consisting of hydrophilic pyridinium salt and a hydrophobic triphenylamino group for labeling CMs and monitoring membrane morphology under different circumstances (Figure 18A) [76]. Probe **18** had negligible fluorescence in aqueous solution due to its high water-solubility induced by the pyridinium salt unit. In the presence of lipid vesicles, however, a clear emission at about 650 nm was observed (Figure 18B). Living cell imaging also revealed that the CM of HeLa cells was specifically lit up by probe **18**. The photostability of probe **18** in HeLa cells by continuous scanning under confocal microscope was studied using CellMask Deep Red CM as the control (Figure 18C). Under the same excitation power, about 20% of probe **18** fluorescent signal was lost after 50 scans, while the fluorescence of the control was almost quenched, showing the high photostability of probe **18**. Using this probe, morphological change in CM in the treatments of Hg^2+^ and trypsin was clearly observed (Figure 18D,E), which indicated that probe **18** was a promising candidate for studying CM-related events.

**Figure 19:**
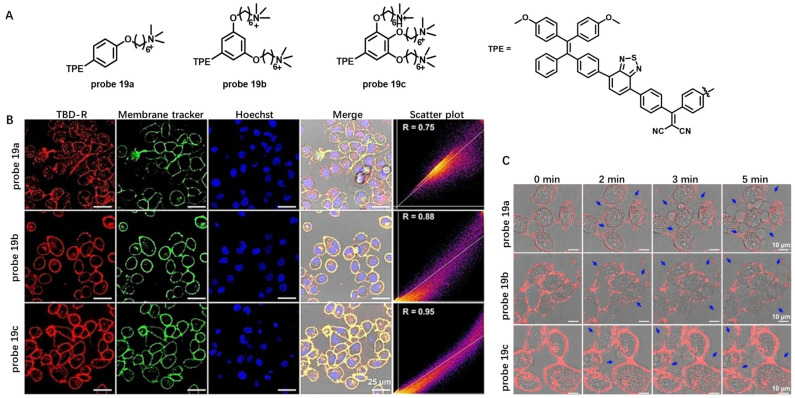
(**A**) Chemical structures of probe **19a**–**c**. (**B**) CM staining using probe **19a**–**c**. (**C**) Real-time observation of probe **19a**–**c** treated HeLa under continuous confocal laser excitation. Scale bar: 10 μm. Copyright 2021 John Wiley and Sons [78].

Initiating pyroptosis is an efficient way to fight against cancer [77]. Recently, Liu et al. developed a series of CM-anchoring AIE photosensitizers, **19a**–**c**, to induce pyroptosis for cancer cell ablation [78]. Probes **19a**–**c** were composed of methoxy-substituted tetraphenylethylene (Donor), benzothiadiazole (Acceptor), phenyl (π), dicyanovinyl (Acceptor) and one, two or three cationic groups (Figure 19A). These probes had weak emission in molecular states and exhibited strong emission at 650 nm in the aggregate states. Different numbers of cationic groups were used to tune their membrane-anchoring ability. Living cell imaging showed that probe **19c** containing three cationic groups had the highest Pearson’s correlation coefficients when compared with probe **19a**–**b** (Figure 19B). Further illumination of the dye-stained cell could induce cell swelling, and CM blew out with bubbles observed by NIR imaging (Figure 19C). This work demonstrated the promising potential of CM anchoring AIE PSs for cancer treatment by activating pyroptosis death pathway.

**Figure 20:**
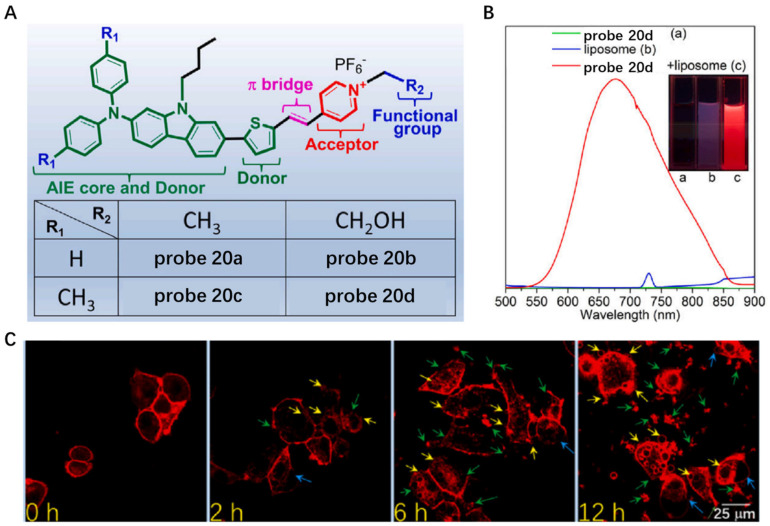
(**A**) Chemical structures of probe **20a**–**d**. (**B**) The fluorescence spectra of probe **20d** in the presence of liposome. (**C**) Realtime monitoring the CM damage using probe **20d**. Scale bar: 25 μm. Copyright 2023 Elsevier [79].

In the following work, a series of novel CM-anchoring NIR AIE photosensitizers (probes **20a**–**d**) were developed by Dai et al. in 2022 (Figure 20A) [79]. These NIR CM staining probes were obtained by introducing the connection electron-accepting (A) units with electron-donating (D) units via π-bridge and extension molecular structure π -conjugation at the same time. In PBS solution, example probe **20c** was weakly emissive, whereas a 285-fold fluorescence enhancement at 677 nm was observed after addition of liposome into the probe solution (Figure 20B), indicating its promising potential of CM anchoring. In addition, the degree of CM damage was observed in real-time when cancer cells were treated with probe **20c** (Figure 20C), which further displayed that this probe could be utilized for photodynamic ablation of cancer cells.

##### Other

Many biomolecules, such as carbon monoxide (CO) and ATP, can function as signaling molecules involved in CM-related events [80,81]. Development of NIR CM-targeting probes that can respond to above biomolecules is significant for studying their roles on CMs.

**Figure 21:**
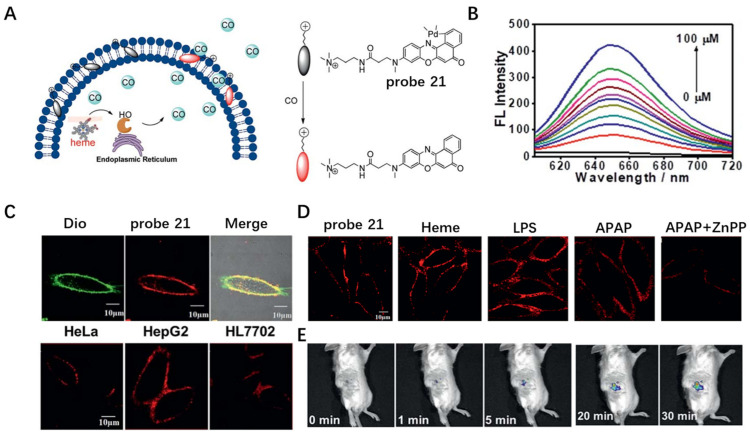
(**A**) Chemical structure of probe **21**. (**B**) The fluorescence response of probe **21** towards CO. (**C**,**D**) Live cell imaging using probe **21** under different conditions. Scale bar: 10 μm. (**E**) In vivo imaging of CO in living mice using probe **21**. Copyright 2019 Royal Society of Chemistry [82].

In 2019, Zhang et al. developed NIR CM-anchored probe **21** to visualize the release behavior of endogenous CO (Figure 21A) [82]. An amphipathic NIR dye was first synthesized by grafting a polar head onto a long and linear hydrophobic Nile Red molecule. Probe **21** was then obtained by complexation of above NIR dye with palladium based on a metal palladium catalyzed reaction. This probe was quenched by the palladium metal atom due to the heavy atom effect. Addition of different concentrations of CO ranging from 0 to 100 μM induced gradual increase in fluorescence intensity at 650 nm in buffer solutions (Figure 21B). Probe **21** exhibited specific CM staining when incubated with HeLa cells. Using this probe, endogenous CO release from normal and cancer cells was monitored and it was observed that the fluorescence intensity on the cancer CM was stronger and more homogeneous than that on the normal CM (Figure 21C). In addition, release of CO from HepG2 cells under LPS and heme-stimulated conditions was verified by the bright fluorescence on CM (Figure 21D). This probe was also used to detect CO in living mice, showing its multifunctional applications.

**Figure 22:**
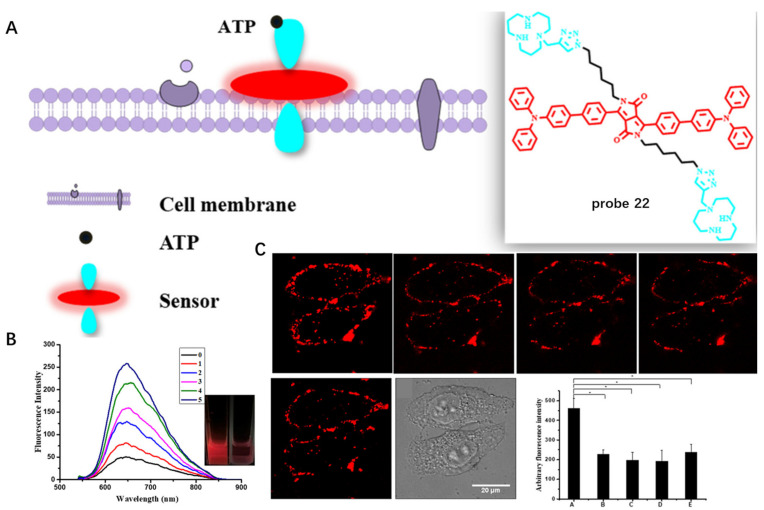
(**A**) Chemical structure of probe **22** and schematic illustration of extracellular ATP detection. (**B**) Fluorescence spec-tra of the probe **22** responding to ATP. (**C**) Fluorescence imaging of HeLa cells with probe **22** for different time. Scale bar: 20 μm. Copyright 2022 Royal Society of Chemistry [83].

Extracellular adenosine triphosphate (ATP) as a signal molecule has significant roles in tumor progression and metastasis. Developing a fluorescent probe to detect extracellular ATP is thus crucial for tumor treatment. In 2022, Yang et al. developed an NIR CM probe **22** with hydrophobic alkyl chains and hydrophilic macrocyclic polyamines for detection of extracellular ATP (Figure 22A) [83]. Upon binding to ATP in buffer solution, probe **22** exhibited enhanced fluorescence by electrostatic interaction and π–π interaction between phosphates and macrocyclic polyamines and adenines and benzene rings with a limit of detection (LOD) of 21 nM (Figure 22B). In addition, with similarity and intermiscibility to the CM, probe **22** was able to specifically target CM and image extracellular ATP (Figure 22C), which provided an efficient method for extracellular ATP detection.

### 2.2. Proteins Targeting Probes

Many proteins with important biological functions are embedded in CMs of live organisms. Therefore, design of CM protein targeting probes or probes that can self-assemble on a CM after being activated by proteins is another efficient way for understanding the physiological processes of CMs.

**Figure 23:**
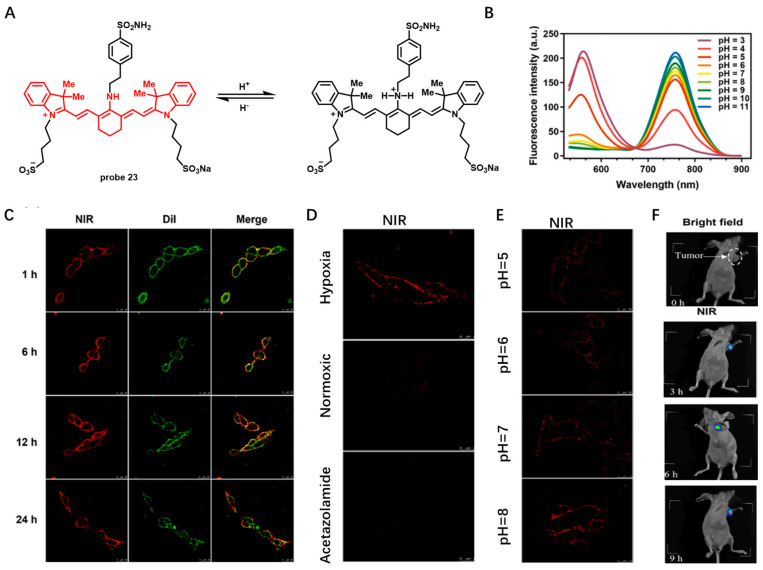
(**A**) Chemical structure of probe **23** and its response to pH. (**B**) Fluorescence emission spectra of probe **23** at different pH values. (**C**–**E**) Long-term CM imaging under different conditions. Scale bar: 25 μm. (**F**) The NIR imaging of tumor mice using probe **23**. Copyright 2023 Elsevier [84].

Carbonic anhydrase IX (CA IX), a transmembrane protein induced under hypoxic conditions, is a promising tumor marker. In 2022, Chen et al. developed pH-sensitive CA IX-targeted NIR probe **23** for fluorescence imaging of hypoxic osteosarcoma (Figure 23A) [84]. This probe was built based on an NIR heptamethine cyanine dye IR783 by connecting with a CA IX targeting group benzenesulfonamide moiety. In buffer solutions, the emission intensity of this probe at 760 nm increased with pH, which was attributed to protonation of amine group under acidic conditions (Figure 23B). After incubation of 143B cells with probe **23** for 1 h or even as long as 24 h, this probe was consistently dispersed on CMs, which showed its excellent long-term CM imaging performance (Figure 23C). CM imaging of 143B cells under simulated normoxia, hypoxia and acetazolamide conditions showed that probe **23** was able to visualize level changes of biomarker CA IX (Figure 23D). Meanwhile, intracellular pH change was also detected by monitoring the fluorescence intensity on CMs (Figure 23E). Moreover, in vivo imaging showed that probe **23** had good selectivity for tumors relative to normal tissues (Figure 23F), indicating its great potential application in biological and medical fields.

**Figure 24:**
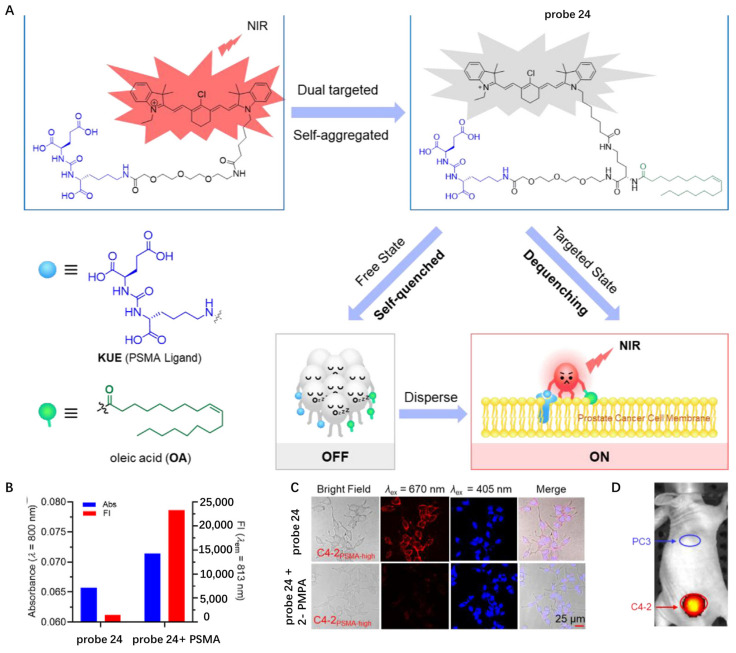
(**A**) Chemical structure of probe **24** and schematic illustration of CM targeting. (**B**) The changes in the absorbance and fluorescence intensity of probe **24** in the presence of PSMA. (**C**) Confocal fluorescent images of C4-2PSMA-high cells with probe **24**. Scale bar: 25 μm. (**D**) In vivo fluorescence images of PSMA-negative and positive tumors using probe **24**. Copyright 2022 Elsevier [85].

For accurate visualization of prostate cancer boundary and lymphatic metastasis, in 2022, Hu et al. developed self-quenched NIR fluorescence probe **24** with dual prostate cancer membrane affinity (Figure 24A) [85]. Glutamate–urea–lysine (KUE)-based moieties were introduced into this probe as a ligand for prostate-specific membrane antigen (PSMA), which was overexpressed in >90% of prostate cancer cells. Oleic acid (OA) motif was further introduced as an additional CM-targeting scaffold to maximize the targeting efficiency of probe **24** for prostate cancer. The dual targeted probe was weakly fluorescent in Tris buffer saline due to a strong self-aggregation effect. Upon adding purified PSMA protein into probe solution, an obvious increase in absorbance and fluorescence was observed, indicating that probe **24** could be activated by PSMA (Figure 24B). Activation of probe **24** by PSMA was also studied in C4-2PSMA-high cells highly expressing PSMA, and the results showed that the CM was specifically lit up, but its signals were erased regarding cells pre-treated with PSMA inhibitor (Figure 24C). In vivo studies revealed that probe **24** could specifically activate in PSMA-positive tumors rather than PSMA-negative tumors (Figure 24D), showing its potential for fluorescence-guided accurate and complete resection of prostate tumors.

**Figure 25:**
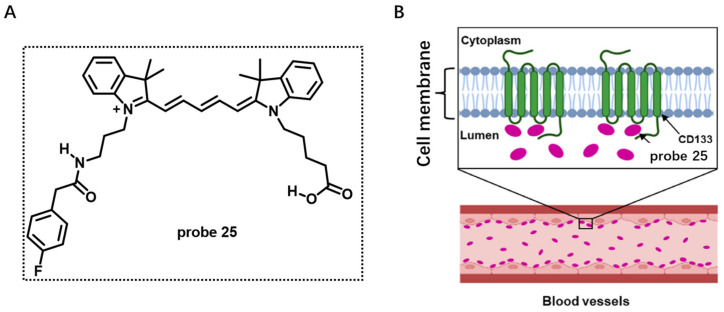
(**A**) Chemical structure of probe **25**. (**B**) The binding site of probe **25** on CD133 protein surface marker of endothelial cells. Copyright 2022 Elsevier [86].

CyA-B2 molecule (probe **25**) was previously selected from library screening for endothelial cells binding (Figure 25A). However, the mechanisms of this probe binding on endothelial cells were unclear. In 2022, by studying the competition assays of CyA-B2 using several potential surface markers of endothelial cells, Matsusaki et al. observed that CD133 provided the lowest IC50 value [86]. Therefore, the CD133 protein expressed on endothelial CM was considered as the binding site of probe **25** due to its suppression on blood capillaries by competition assays (Figure 25B). Since CD133 is expressed on many types of cancer cells, there would be great potential to use this probe as a bioprobe to monitor or diagnose tumor growth.

**Figure 26:**
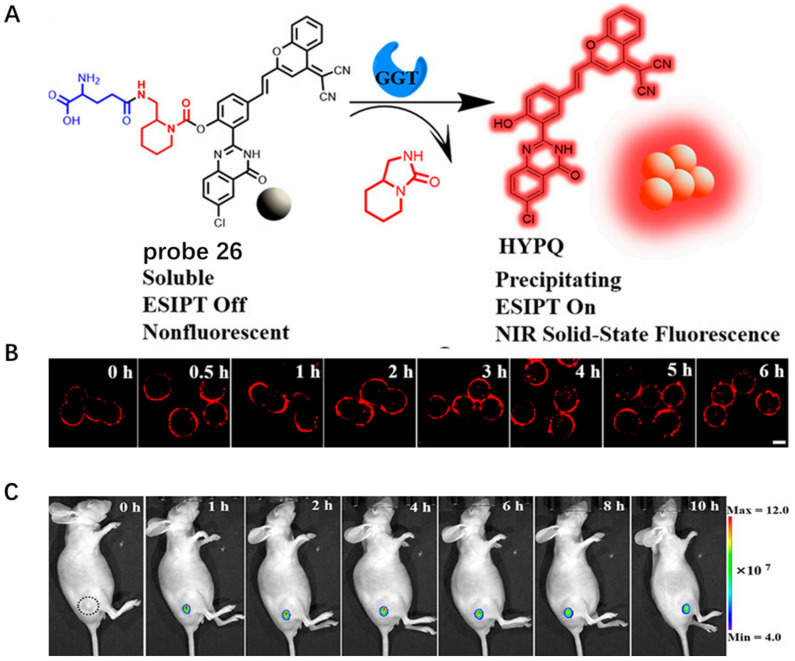
(**A**) Chemical structure of probe **26** and its response to GGT. (**B**) Long-term in situ im-ages of probe **26** in live cells. Scale bar: 20 μm. (**C**) Long-term in situ imaging of GGT in tumors using probe **26**. Copyright 2021 National Academy of Sciences [87].

Conventional lipid-conjugated amphiphilicity CM probes usually face the problem of gradually diffusing into the cell from the CM surface after a certain period of time, thereby losing in situ information in long-term bioimaging. In 2021, Zhang et al. developed antidiffusion NIR probe **26** based on a fluorochrome HYPQ characterized by strong hydrophobicity and low lipophilicity to address this challenge using γ-glutamyltranspeptidase (GGT) as an example (Figure 26A) [87]. HYPQ was designed by conjugating strong hydrophobic solid-state fluorochrome 6-chloro–2-(2-hydroxyphenyl) quinazolin-4(3H)-one (HPQ) with a 2-(2-methyl–4H-chromen–4-ylidene) malononitrile group. After probe **26** was activated by GGT, the fluorescence signal on the CM remained unchanged, even with incubation time extending to 6 h, which was attributed to the precipitating and stable signal properties of HYPQ and was significant for in situ monitoring of GGT activity (Figure 26B). In vivo imaging revealed that probe **26** could accurately define tumor regions after long-term in situ imaging of tumor-bearing mice (Figure 26C). The excellent performance of HYPQ makes it an ideal alternative to construction of universal antidiffusion fluorescent probes and provides an efficient method for accurate imaging-guided surgery in the future.

**Figure 27:**
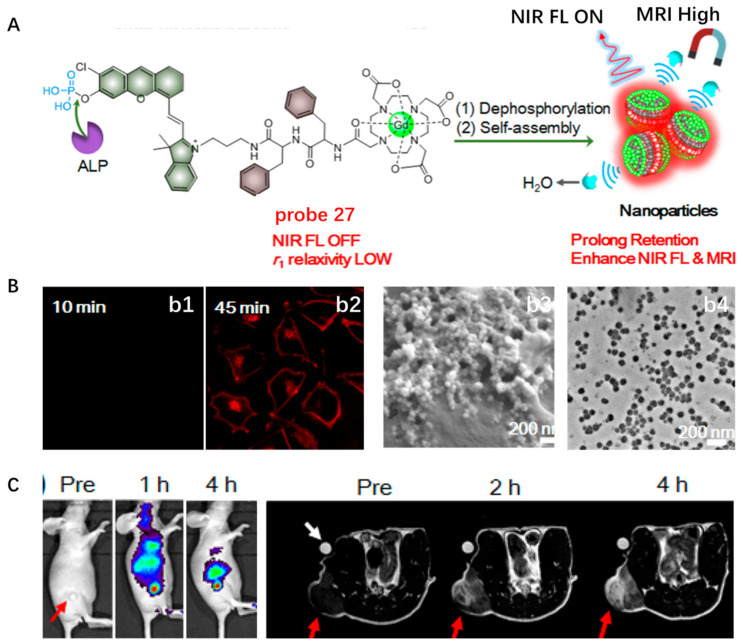
(**A**) Chemical structure of probe **27** and ALP-mediated in situ self-assembly into NPs. (**B**) NIR fluorescence im-aging of HeLa cells incubated with probe **27** ((**b1**,**b2**), Scale bar: 25 μm) and cryo-SEM image and TEM image of the self-assembled NPs on CM ((**b3**,**b4**), Scale bar: 100 μm.). (**C**) NIR fluorescence and MRI imaging of endogenous ALP in tumor. Copyright 2019 American Chemical Society [88].

Another stimuli-responsive in situ self-assembly of NIR small molecules on the surface of CM was reported by Ye et al. in 2019 (Figure 27) [88]. Probe **27** consisted of a pre-quenched NIR fluorophore caged by an ALP recognition phosphate group, a paramagnetic DOTA-Gd chelate for MRI and a hydrophobic dipeptide Phe-Phe (FF) linker to promote self-assembly (Figure 27A). This probe had high water solubility, weak NIR fluorescence and low r1 relaxivity. After HeLa cells were incubated with probe **27**, bright NIR fluorescence was observed on the CM where ALP tended to locate (Figure 27B). In situ self-assembled NPs anchored on the HeLa CM were detected with cryo-SEM and TEM. This probe with activatable NIR fluorescence and MRI via in situ self-assembly was suitable for noninvasively measuring and localizing ALP activity in live tumor cells and living mice (Figure 27C).

## 3. Conclusions

A CM is a phospholipid bilayer involved in various cellular activities. In this review, we summarized development of small-molecule-based CM-targeting fluorescent probes with NIR spectra from aspects of design principles, CM-targeting mechanisms and biological applications. Designing amphipathic probes that have similarity and intermiscibility to a CM is an effective method to realize CM imaging. The regular method for development of such probes is to incorporate positively or negatively charged groups to lipophilic dyes. Moreover, in order to prolong retention of these probes on a CM, long alkyl chains are adopted to assist probe insertion into the CM by hydrophobic interaction with the alkyl chain of a phospholipid. Many CM-targeting probes have always-on fluorescence before and after being anchored on a CM, which requires a repeated washing procedure and prolonged incubation. Currently, several approaches have been used to construct activatable CM staining probes, including an environmentally sensitive fluorescent probe based on solvatochromic and fluorogenic dyes, AIE-based fluorescent probes and disassembly light-up fluorescence strategy. These probes enable in situ CM imaging with high signal-to-noise ratio in a washing-free manner. Designing probes that can target CM proteins or be activated by these proteins to form self-assembly on CMs is another effective way to realize CM imaging, and several such probes are overviewed and discussed in this review.

Based on the above discussion, we believe that activatable NIR CM probes with advantages of low background interference and high signal-to-noise ratio deserve pursuing. In this process, several key parameters, including dosage, response time, retention time and biocompatibility, should be carefully evaluated. Ideal activatable NIR CM probes may have properties of high solubility in cultural medium with quenched fluorescence, fast signal output, high signal-to-noise ratio, strong CM-targeting ability and low cytotoxicity. In order to achieve such probes, a CM-targeting group with strong targeting ability, dyes with capability of easy quenching and signal recovery and mechanism of probe interaction with CMs should be explored. Moreover, the principles of designing such probes with easy synthesis and good biocompatibility should also be thoroughly studied. Visualization of the nanoscale organization of CMs is very important but a challenging task. Probes that can be applied for super-resolution imaging of CM organization will be highly pursued in the future. Moreover, the field of designing multifunctional probes with CM imaging and phototherapy effect is worth further exploration.

## Data Availability

Not applicable.

## Sample Availability

Samples of the compounds (probe **1**–**27**) are available from the authors.
